# Multiparametric Orthogonal Characterization of Extracellular Vesicles by Liquid Chromatography Combined with In-Line Light Scattering and Fluorescence Detection

**DOI:** 10.1021/acs.analchem.3c02108

**Published:** 2023-08-09

**Authors:** Karl Normak, Marcell Papp, Michael Ullmann, Carolina Paganini, Mauro Manno, Antonella Bongiovanni, Paolo Bergese, Paolo Arosio

**Affiliations:** † Department of Chemistry and Applied Biosciences, ETH Zurich, Zurich 8093, Switzerland; ‡ Institute of Biophysics, National Research Council of Italy, Via Ugo la Malfa 153, Palermo 90146, Italy; § Institute for Research and Biomedical Innovation (IRIB), National Research Council of Italy, Via Ugo La Malfa 153, Palermo 90146, Italy; ∥ Department of Molecular and Translational Medicine, University of Brescia, Brescia 25123, Italy; ⊥ Center for Colloid and Surface Science (CSGI), Florence 50019, Italy

## Abstract

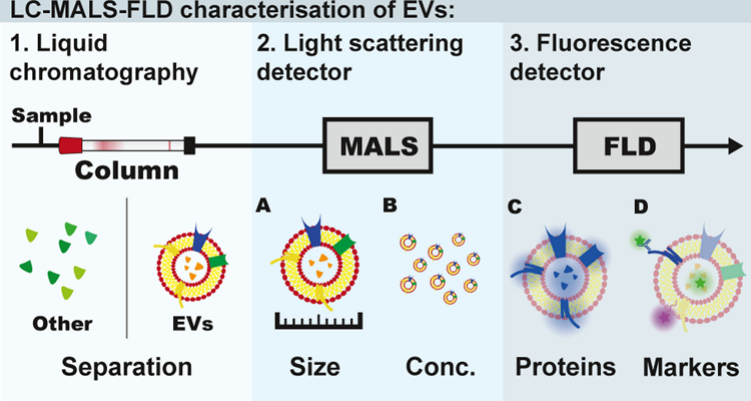

Extracellular vesicles (EVs) are membrane-enclosed biological nanoparticles with potential as diagnostic markers and carriers for therapeutics. Characterization of EVs poses severe challenges due to their complex structure and composition, requiring the combination of orthogonal analytical techniques. Here, we demonstrate how liquid chromatography combined with multi-angle light scattering (MALS) and fluorescence detection in one single apparatus can provide multiparametric characterization of EV samples, including concentration of particles, average diameter of the particles, protein amount to particle number ratio, presence of EV surface markers and lipids, EV shape, and sample purity. The method requires a small amount of sample of approximately 10^7^ EVs, limited handling of the sample and data analysis time in the order of minutes; it is fully automatable and can be applied to both crude and purified samples.

## Introduction

Extracellular vesicles (EVs) are membrane-enclosed biological nanoparticles secreted by all cells.^1^ These vesicles mediate intercellular communications and play a role in both functional and dysfunctional biology. They are capable of crossing biological barriers, delivering important biomolecules, such as proteins, nucleic acids, lipids, and metabolites, and evoking response in recipient cells.^2^ Due to these properties, EVs have been heavily investigated as diagnostic marker carriers^3,4^ and as a therapeutic vehicle for drug and gene delivery,^5,6^ or as EV-based therapeutics per se.^7^

The complex and heterogeneous nature of EVs poses challenges for their analytical characterization, which requires multiparameter analysis.^2^ MISEV2018 guidelines recommend describing: (i) size and concentration of particles, (ii) protein amount, (iii) EV markers, and (iv) lack of contaminants.^8^ Combining multiple characteristics into ratios is recommended as a more robust indicator of purity. This level of comprehensive characterization can be only achieved by combining multiple different analytical methods that require different instruments, significant hands-on time, or different degrees of sample preparation.

The study of EVs has progressed from bulk analysis toward tools with single-particle resolution. These methods enable the analysis of specific subpopulations and are crucial to address the heterogeneous nature of EV samples.

However, in some contexts, there is the need of high-throughput techniques that require limited sample preparation. This is the case, for instance, of quality control in large-scale manufacturing,^5,9,10^ where a compromise between multiparametric analysis, limited sample handling, and compatibility with crude samples is required.

We have previously shown that a microfluidic platform based on the combination of diffusion sizing and multiple wavelength fluorescence detection allows us to get complementary information such as average size, concentration, presence of biomarkers, and purity of EVs.^11^ The core of the approach relies on the multiparametric analysis of the sample on the same platform.

Here, we show that liquid chromatography combined with an in-line multi-angle light scattering detector (MALS) and fluorescence detector (FLD) in one single apparatus can provide multiple information on EV samples, such as concentration, morphology, and average size of the particles, protein-to-particle ratio, presence of EV surface markers and lipids, and purity.

**Figure 1:**
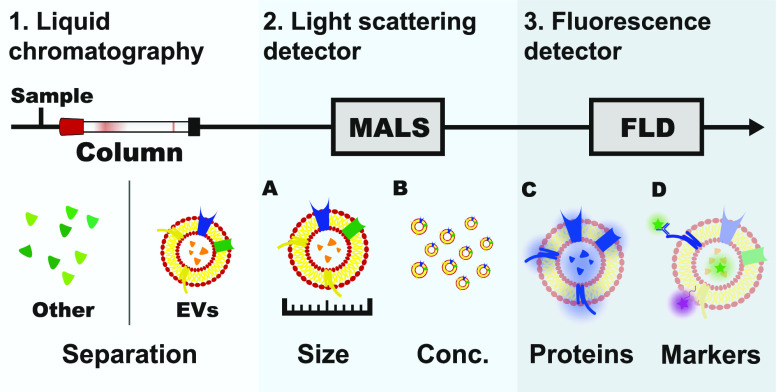
Schematic representation of the analytical system. Samples were injected into the chromatographic column (either size exclusion or ion exchange) to separate different species. The eluate was analyzed first by an in-line multi-angle light scattering (MALS) detector and then by a multiple wavelength fluorescence detector (FLD).

A schematic representation of the main components of the system is shown in Figure 1. The components are connected together into one single unit by capillary tubing, therefore eliminating the need for any manual sample handling between the steps. In this modular setup, the analyzed vesicles are first separated from other co-isolates by liquid chromatography; the size and concentration of particles is then analyzed by a light scattering detector and, finally, a fluorescence detector measures the intrinsic fluorescence of proteins and the fluorescence signal of specific markers. This comprehensive characterization of structure and composition requires minimal hands-on preparation time and limited amounts of EVs in the order of 10^7^ particles and provides the majority of necessary information required by the MISEV2018 guidelines. Moreover, coupling light scattering and fluorescence detection with liquid chromatography allows us to analyze both crude and purified samples by separating the vesicles from the other components present in the mixture.

## Experimental Procedure

### Extracellular Vesicles

EVs were produced according to a previously published method.^12,13^ In brief, HEK293-F cells (Thermo Fisher) were cultured at 37 °C CD293 medium (Thermo Fisher) supplemented with 4 mM GlutaMAX and 250 mg/L Pluronic F-68 (Thermo Fisher). The culture was stirred at 250 rpm and maintained at pH 7.1 and a dissolved oxygen concentration of 40% in a stirred tank bioreactor (DASGIP, Eppendorf) for 136 h. Conditioned media (1 L) was harvested from 1.8 × 10^8^ cells with 92% viability and clarified by two centrifugation steps, the first at 200*g* for 10 min and the second at 3000*g* for 15 min, and frozen in 50 mL aliquots. The aliquots were thawed and filtered by a 0.22 μm PES syringe filter (TPP, Switzerland). To remove DNA and other nucleotide contaminations, 100 U of Pierce nuclease (Thermo Fisher) was added to the solution. The sample was then concentrated using an Amicon 50 kDa MWCO ultrafilter (Merck Millipore, Ireland). Aggregates were removed by centrifugation at 7000*g* for 5 min before separation by size exclusion chromatography (SEC) using a Sepharose CL-4B resin (Sigma-Aldrich, Germany) packed in a Econo-Pac (Bio-Rad) gravity flow column. The collected vesicle fractions were combined, and the concentration was measured by nanoparticle tracking analysis (NTA), and surface markers were characterized by flow cytometry and the morphology by electron microscopy (EM).

### Nanoparticle Tracking Analysis (NTA)

Measurements were performed on a Zetaview NTA (Particle Metrix, Germany). Before each series of measurements, the system was calibrated using polystyrene beads following the manufacturer’s instructions. Samples were diluted in PBS (Thermo Fisher) (typically 100–1000 fold) to achieve low concentrations suitable for NTA measurements. For the comparison of MALS results with NTA data, 1 mL of the sample fractionated by SEC (0.5–1.5 mL elution volume) was directly analyzed by NTA without further dilution. The sensitivity was set to 85 and the shutter to 125. For each sample, 11 positions were considered for analysis. All measurements were performed in triplicate, and the average was evaluated.

### Dynamic Light Scattering (DLS)

The size of HEK293F-derived EVs was measured using a Zetasizer Nano ZSP system (Malvern Panalytical, UK) working in backscattering mode at 173° at 25 °C. The sample was diluted in PBS (Thermo Fisher).

### Cryo-EM Imaging

For cryo-EM analysis, 3.4 μL of sample at 10^11^ Particles/mL was applied onto Quantifoil R2/1 300 mesh copper grids, which were previously negatively glow-discharged at 25 mA for 30 s with a PELCO easiGlow (Ted Pella) glow discharge cleaning system. Excess of the sample was blotted away for 2 s and plunge-frozen in a liquid ethane/propane mixture (continuously cooled by liquid nitrogen) using Vitrobot Mark IV (Thermo Fisher) with an environmental chamber set to 100% humidity and 22 °C.

Vitrified grids were observed under Titan Krios (Thermo Fischer) electron microscope using a K2 camera fitted behind a Bio Quantum energy filter from Gatan. K2 zero-loss filtered integrated micrographs representing a cumulative dose of ∼45 electrons per Å^2^ were collected at approx. −3 μm defocus and 1 Å pixel size.

### Flow Cytometry

5 μL of exosome-human CD81 flow detection reagent (Thermo Fisher) was washed in 200 μL of 0.1% BSA in PBS. 10 μL of sample was diluted to a final concentration of 0.1% BSA and a volume of 100 μL. The sample was added to the washed beads and incubated overnight at 4 °C while shaking. The beads were washed 2× with 300 μL of 0.1% BSA before resuspending in 100 μL 0.1% BSA in PBS. 1.2 μL of anti-human CD81-APC (Thermo Fisher), anti-human CD63-PE (Thermo Fisher), anti-human CD9-FITC (Thermo Fisher) conjugate, or the corresponding antibody isotype controls (Thermo Fisher) were added to each sample and shaken protected from light for 1.5 h. After additional two washing steps, the beads were resuspended in 150 μL PBS with 0.1% BSA.

The flow cytometry measurements were performed on a Beckman Coulter (USA) CytoFLEX S flow cytometer. The gain of the forward scattering was set to 199, the side scattering to 48, the FITC (525/40 nm bandpass filter and blue laser), APC (660/10 nm bandpass filter and red laser) and PE (585/42 nm bandpass filter and yellow laser) channel to 3000. Before measuring each sample, the plate was mixed for 3 s. Measurements were recorded for 180 s. Between each sample, 2 injections of water were performed for 30 s to clean the line. The data were analyzed using a custom-made Python program. The gating thresholds for the scattering signals were set based on the flow cytometry events of the flow detection reagent. Single bead events with a side scattering intensity between 350 000 and 175 000 and a forward scattering intensity between 65 000 and 250 000 were selected for analysis.

### Size Exclusion Chromatography (SEC)

20 μL of purified EVs diluted to 10^10^ particles/mL in PBS (unless otherwise specified), or 20 μL of conditioned media was injected into a Tricorn-5/100 column (Cytiva) of 2 mL bed volume packed with Sepharose-CL4B resin (Sigma-Aldrich, Germany) assembled on an Agilent 1200 Series HPLC system. Phosphate-buffered saline (PBS) (137 mM NaCl, 2.7 mM KCl, 10 mM Na_2_HPO_4_, 1.8 mM KH_2_PO_4_) at a volumetric flow rate of 0.1 mL/min was used as an eluent.

### Anion Exchange Chromatography (AIEX)

40 μL of purified EVs diluted to 10^10^ particles/mL in PBS was injected into a 1 mL CIMmultus EV column (BIASeparations, Slovenia) assembled on an Agilent 1200 Series HPLC system. 50 mM HEPES at pH 7.4 with 890 mM NaCl at a volumetric flow rate of 1 mL/min was used as the binding and washing buffer. EVs were eluted after 15 mL of binding and washing buffer in 50 mM HEPES at pH 7.4 with 2 M NaCl at a volumetric flow rate of 1 mL/min.

### EV Characterization by Fluorescence Spectroscopy

All stock solutions of different fluorescent stains were diluted in PBS containing 10^10^ particles/mL of EVs. Anti-human CD81-AlexaFlour488 antibody (R&D Systems) was diluted 1:9999 from stock. 1,1′-Dioctadecyl-3,3,3′,3′-tetramethylindocarbocyanine perchlorate (DiI) (Chemodex, Switzerland) was diluted from the stock solution of 1 mM in ethanol to 10 μM in PBS and incubated with EVs for 4–6 h at room temperature. CalceinAM (Invitrogen) was diluted from 1 mM in DMSO 10 μM in PBS and incubated with EVs overnight at room temperature.

The fluorescence signal was measured with an Agilent 1260 Infinity II fluorescence detector assembled after the chromatographic column. Excitation and emission wavelengths were set to λ_ex_ = 280 nm and λ_em_ = 350 nm for intrinsic fluorescence, λ_ex_ = 480 nm and λ_em_ = 520 nm for anti-human CD81-AlexaFlour488 and CalceinAM, and λ_ex_ = 565 nm and λ_em_ = 595 nm for DiI. Fluorescence gain was set to 14, except for anti-human CD81-AlexaFlour488 quantification, where it was set to 16. Raw data were exported from the ChemStation software, baseline corrected and integrated using a Python script. Protein amount was calculated using a calibration curve obtained with BSA (Sigma-Aldrich, Germany) samples at known concentrations.

### Particle Quantification by Multi-Angle Light Scattering (MALS)

Multi-angle light scattering (MALS) analysis was performed with an in-line DAWN HELEOS II detector (Wyatt Technology). The system was calibrated using toluene, and detectors were normalized using bovine serum albumin (Sigma-Aldrich, Germany) as an isotropic scatterer standard. The laser wavelength was set to 658 nm. Rayleigh ratio data were collected by ASTRA 5 or ASTRA 7 Software (Wyatt Technology). Rayleigh ratio data were normalized, despiked using a median filter, smoothed by Savitzky-Golay filtering, and baseline corrected to obtain the excess Rayleigh ratios (*R*(θ)).

## Results and Discussion

### EVs Prepared from HEK293F Cells

**Figure 2:**
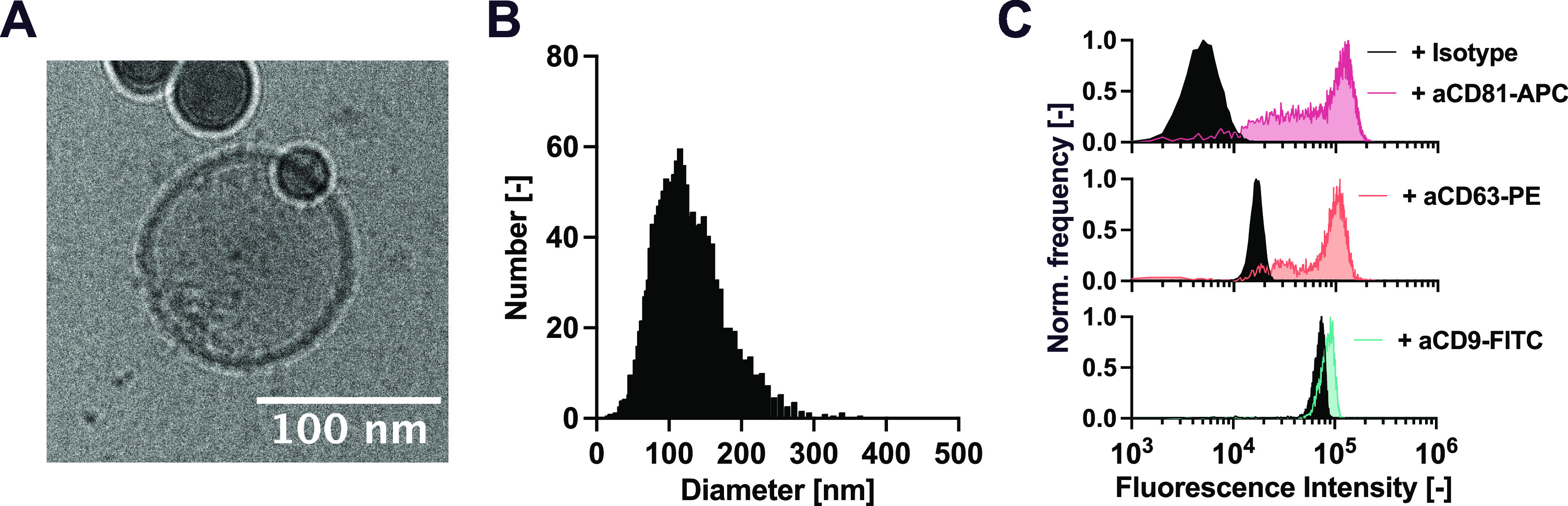
(A–C) Characterization of EVs derived from HEK293F cells: (A) Cryo-TEM image of purified EVs. (B) Size distribution of the EVs measured by NTA. (C) Presence of common EV surface markers CD81, CD63, and CD9 measured by bead-based flow cytometry.

We produced extracellular vesicles from HEK293F cells according to a method previously described (see the Experimental Procedure section).^12,13^ We characterized the vesicles using a combination of cryo-transmission electron microscopy, nanoparticle tracking analysis, and flow cytometry. The particles exhibited the characteristic circular bilayer structure (Figure 2A) and size distribution in the range from 50 to 300 nm (Figure 2B). Flow cytometry analysis confirmed the presence of the common EV biomarkers CD81, CD63, and CD9 (Figure 2C).^14,15^

### Size and Concentration of Particles from Multi-Angle Light Scattering

First, we aimed to characterize the size and concentration of particles in the EV sample by injecting 20 μL (containing 2 × 10^8^ particles as measured by NTA) into a SEC column coupled with an in-line light scattering detector.

**Figure 3:**
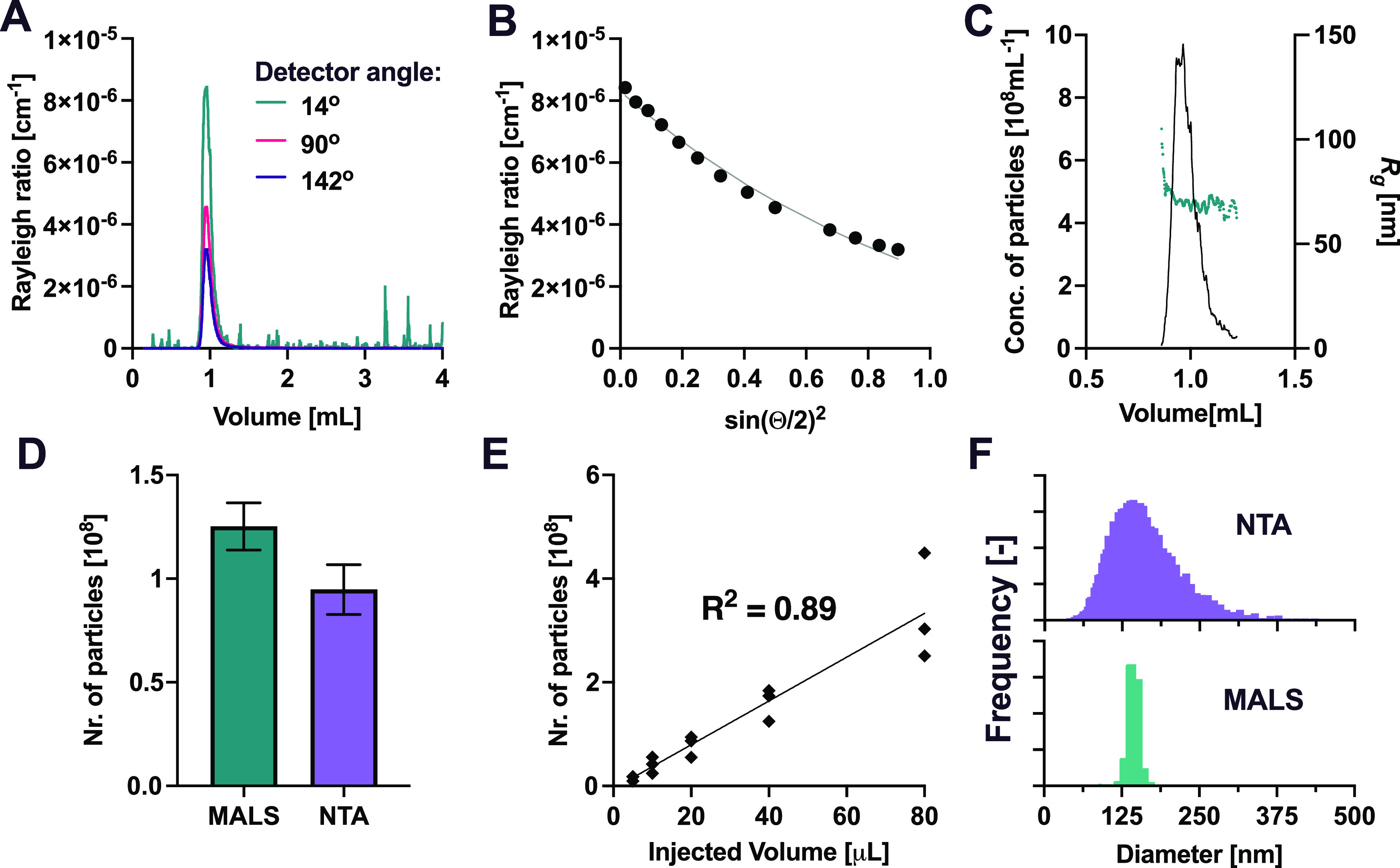
MALS characterization of EVs. (A) Scattered light intensity (Rayleigh ratio) measured at different elution volumes at selected angles (14, 90, and 142°). (B) Scattered light intensity (Rayleigh ratio) measured at different angles θ (black dots) at 0.95 mL elution volume. The continuous line represents the fit according to eqs 1 and 2 and provides the radius of gyration *r*. (C) Radius of gyration (green dots) and concentration of particles measured according to eq 3 (black line) at different elution volumes. (D) Comparison between the total number of particles measured by integrating the SEC-MALS data shown in panel (C) and by NTA (see Experimental Procedure). (E) Number of particles measured by the SEC-MALS system upon progressive decrease of the injection volume. (F) Comparison between different EV sizing techniques based on light scattering. Diameter distribution of EV samples measured by SEC-MALS and NTA.

At each elution time, the MALS detector measures the intensity of scattered light at different scattering angles θ. The scattered intensity is expressed as the Rayleigh ratio *R*(θ) (Figure 3A,B) and is proportional to the amount and size of the particles according to eq 1
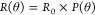
1
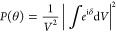
where *R*_0_ is the excess Rayleigh ratio at 0° angle and *P*(θ) is the form factor, which describes how the scattered light intensity at angle θ is modified due to the shape of the particle with volume *V* and radius of gyration *R*_g_. We considered the effective form factor *P*′(θ) of a spherical shell (eq 2) with a volume of sphere with the radius *b*(16,17) and with a shell thickness *m* of 10 nm, and an inner radius *a* = *b* – *m*
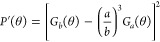
2
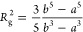
*for m/b* ≪ 1: *R*_g_ ∼ *r*
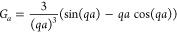

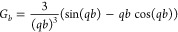

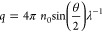
where *a* and *b* are, respectively, the inner and outer radius of the shell, *G*_a_ and *G*_b_ are, respectively, the shell’s inner and outer scattering functions, λ the laser wavelength (658 nm), and *n*_0_ the refractive index of the solution (1.33 for PBS and 1.352 for 50 mM HEPES buffer with 2 M NaCl^18,19^). The radius of gyration *R*_g_ can be approximated by *r* if the shell thickness is significantly smaller than the diameter of the particle (10 nm/140 nm ≈ 0.07 ≪ 1). By fitting the measured *R*(θ) at different angles (Figure 3B) with simulations based on eqs 1 and 2, we estimated *R*_0_ and the radius *r* of the particles at each elution time.

The concentration of particles *c* can be evaluated from first principles by considering the Rayleigh ratio *R*(θ) and the differential scattering function of an individual particle *i*(θ)^20,21^ at any angle according to eq 3(22)
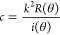
3
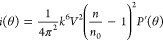

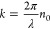
Where *V* is the particle volume and *n* the refractive index of the analyzed particle. Assuming a spherical shape, the particle volume *V* was calculated from the outer radius (*b*) estimated previously. The effective refractive index of EVs (*n* = 1.41)^23,24^ was obtained from the literature.

The concentration of particles can be calculated at any scattering angle.^16,22^ We calculated *c* using the scattering intensity at 0° angle (*R*_0_) estimated previously. The derivation for eq 3 is shown in the Supporting Information.

We note that this approach based on MALS analysis quantifies particles without requiring a calibration curve.

The MALS signal was recorded for infinitesimal fractions of samples eluting every 2 s from the column. For each eluting fraction, the particle concentration and size were calculated (Figure 3C).

The total number of particles was obtained by integrating EV concentration over elution time from 0.7 to 1.2 mL (Figure 3C). To validate the particle quantification by the MALS method, 1 mL fraction of EVs eluting from the column was collected and analyzed offline by NTA. The results of MALS and NTA were very consistent in terms of the total number of particles measured (Figure 3D). The MALS method yielded a slightly larger number of particles, likely due to the low separation resolution and the obscuration of smaller particles by larger particles.

We injected a series of EV samples at different injection volumes to progressively decrease the number of particles and verified that the number and concentration measured by MALS was proportional to the expected value (Figure 3E). The particle numbers provided by MALS scaled linearly with the injection volume in a wide particle number range. A lower limit of quantification of at least 10^7^ particles was observed.

From the MALS data (Figure 3A–C), a weighted size distribution was calculated from the outer radius *b* (see above), and the concentration of particles was measured at each elution time and compared with the results obtained by nanoparticle tracking analysis (NTA) (Figure 3F). The average size was further evaluated by bulk dynamic light scattering (DLS).

The size distribution from MALS was narrower compared to NTA due to the poor separation of particles in the 50–200 nm size range by SEC. Clearly, the single-particle NTA analysis provides a more realistic representation of the EV size distribution. The average diameter of the size distribution measured by MALS was consistent with both NTA and DLS (148 nm for all techniques, see also Supporting Figure S2). We note that the techniques are based on different principles and measure two different sizes: while NTA and DLS measure diffusion coefficients and therefore report on the hydrodynamic radius, MALS evaluates the radius of gyration.

### Protein Amount to Particle Number Ratio

**Figure 4:**
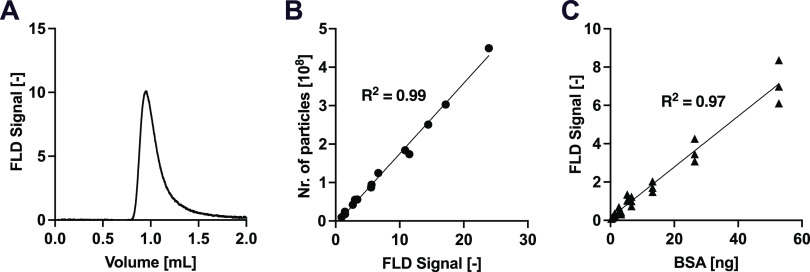
Characterization of EV total protein amount using intrinsic fluorescence of tryptophan residues. (A) Intrinsic fluorescence signal of an EV sample injected into the SEC column (same sample as in Figure 2A). (B) Correlation between the number of vesicles measured by MALS and the intrinsic fluorescence signal at decreasing EV sample injection volume. (C) Calibration curve of the intrinsic fluorescence signal of BSA vs injected BSA amount.

The results described in the previous paragraph showed the application of SEC-MALS to evaluate EV size and concentration from the angular dependence and intensity of the light scattering. The SEC-MALS system can be further coupled with an in-line fluorescence detector (FLD) to measure the total protein amount of EVs (Figure 4).

The total protein amount of the particles eluting from the column can be measured by monitoring the intrinsic fluorescence signal of the tryptophan residues. A representative example is shown in Figure 4A, which reports the tryptophan fluorescence signal corresponding to EVs eluting at 1 mL volume (see also MALS signal in Figure 3A). For the EV samples analyzed at decreasing injection volumes, as expected, the number of vesicles measured by MALS correlated linearly with the intrinsic fluorescence signal (Figure 4B). By using a calibration curve based on BSA samples of known concentrations (Figure 4C), the total amount of protein can be quantified from the measured fluorescence signal. The analyzed EVs contained 4.2 × 10^–7^ ng protein/particle, which is well in agreement with previous calculations.^25^

The information from the FLD can be combined with the particle concentration measured by MALS to characterize the ratio of protein amount to particle number, which is an important property to describe sample purity, as recommended by the MISEV2018 guideline.

### EV Composition, Markers, and Morphology

In addition to the total protein content, the application of an in-line fluorescence detector allows to evaluate EV identity by quantifying the amount of specific markers and different components of EVs upon suitable staining. An advantage of combining the fluorescence detection with chromatography is the removal of the unbound label from the labeled EVs.

**Figure 5:**
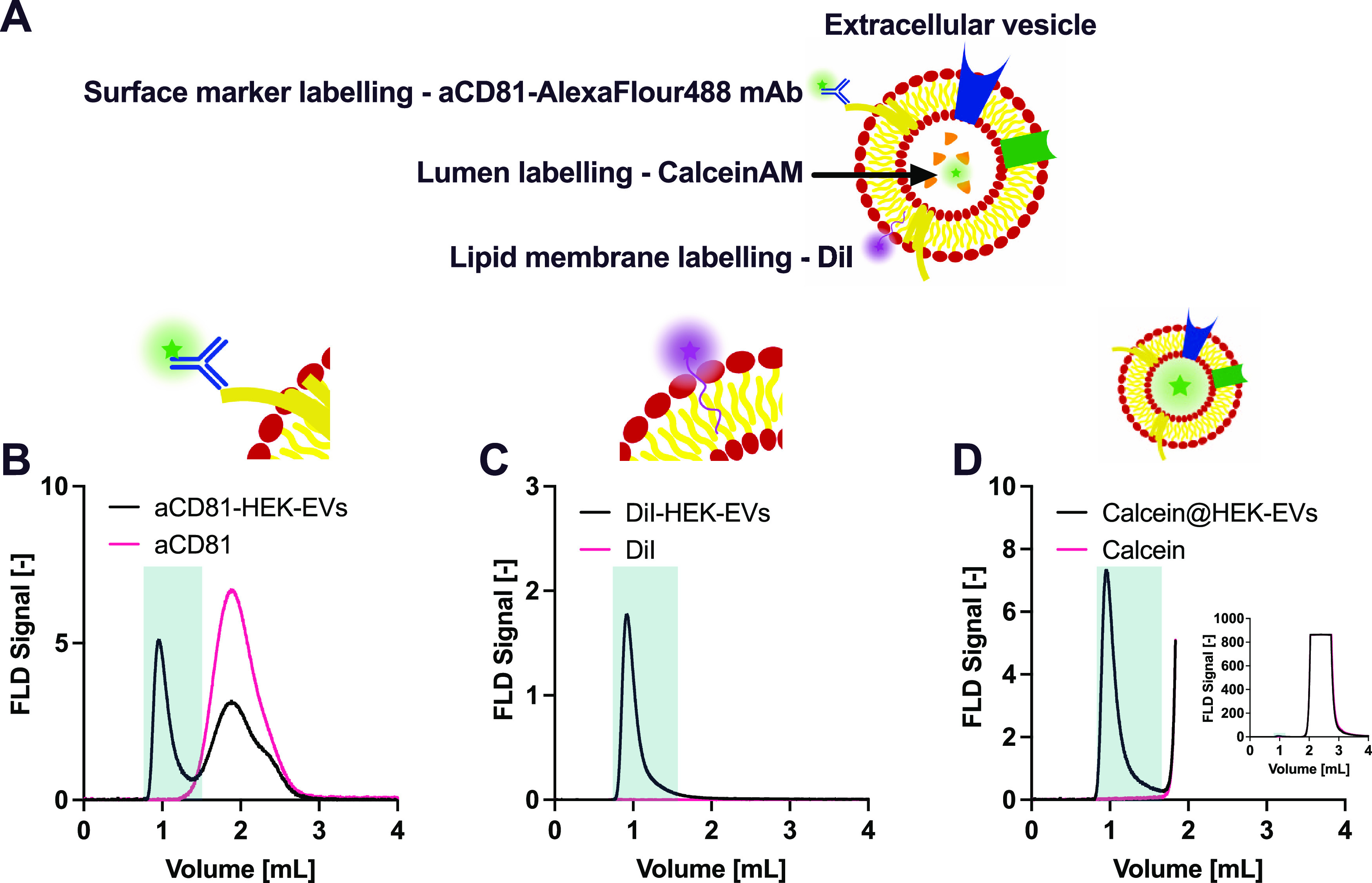
Characterization of vesicle markers by fluorescence detection. (A) Illustration of the strategy for the fluorescent labeling of EVs. (B–D) Chromatograms of anti-CD81 antibody (B), DiI (C), and CalceinAM (D) in the absence (red) and presence (black) of EVs. In panel (D), the data are plotted until 1.8 mL for clarity. The inset shows the full chromatogram. The eluted fluorescently labeled EV fraction is highlighted in green.

Here, three labeling strategies were used to quantify different components of EVs (Figure 5A):

(i). The surface biomarker CD81 was stained with a fluorescently labeled anti-human CD81 antibody conjugated to an AlexaFluor488 dye, which confirmed that the EV particles were CD81 positive (Figure 5B). The elution volume of fluorescent EV was approximately 1 mL, while the free antibody eluted later.
(ii). The lipid membrane was labeled with the lipophilic dye DiI (Figure 5C), which becomes fluorescent when incorporated in a phospholipid membrane. Control experiments showed that the dye did not form fluorescent particles of EV size in our tested conditions. In general, care should be taken when applying lipophilic carbocyanine dyes since they may potentially form particles with sizes similar to EVs.^26,27^ The problem could be mitigated by using photoinducible dyes such as a silicon-containing rhodamine diazoindanone derivative.^11,28^ In this case, the free dye does not exhibit any fluorescence and therefore dye particles do not perturb the analysis.
(iii). The EV lumen was stained with the membrane-permeable dye CalceinAM. After hydrolysis, this dye becomes membrane impermeable and remains trapped in the lumen of the vesicle. The particles eluting at 1 mL volume were positive for CalceinAM (Figure 5D), indicating that the analyzed particles have an intact membrane shell.

Altogether, the SEC-MALS-FLD analysis shows that the particles eluting at 1 mL have an average diameter of 148 nm, have a protein-to-particle ratio of 4.2 × 10^–7^ ng protein/particle, have a shell structure, contain lipids, and are CD81 positive. The combined information on size, morphology, and composition identifies these particles as EVs.

### Characterization of EVs from Crude Samples and EV Purity

The previous paragraphs demonstrated the utility of liquid chromatography coupled with MALS and FLD for the characterization of various parameters of EVs. The analysis was performed on EVs that had been previously purified from more complex media.

**Figure 6:**
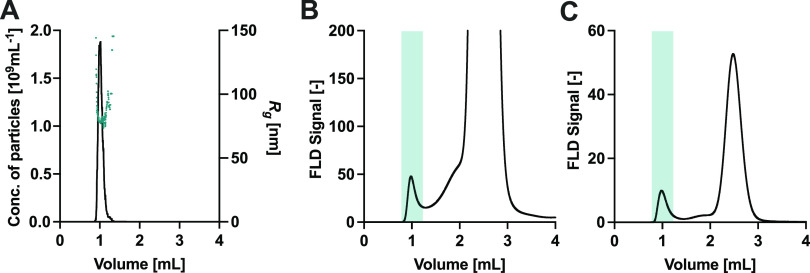
Characterization of vesicles directly from conditioned media by using size exclusion chromatography coupled to light scattering and fluorescence detectors. The eluted EV fraction is highlighted in green. (A) Number of particles and their radius. (B) Native fluorescence signal and (C) anti-human CD81 signal.

We applied the in-line MALS-FLD method to analyze EVs directly from conditioned media. SEC effectively separated the EV particles from the rest of the media as shown by the main peak eluting at 1 mL in Figure 6A, which contains a large number of particles with an average diameter of 172 nm. This peak exhibited an intrinsic protein fluorescence signal (Figure 6B) and contained species, which could be stained with an anti-human CD81 antibody (Figure 6C).

Moreover, the in-line MALS-FLD detection can provide information about sample purity when used in combination with other separation methods that are complementary to SEC, such as ion exchange chromatography^26,27^ and asymmetric field-flow fractionation.^28,29^ This combination of multiple, complementary separation modes enables the investigation of the presence of contaminants in the sample.

**Figure 7:**
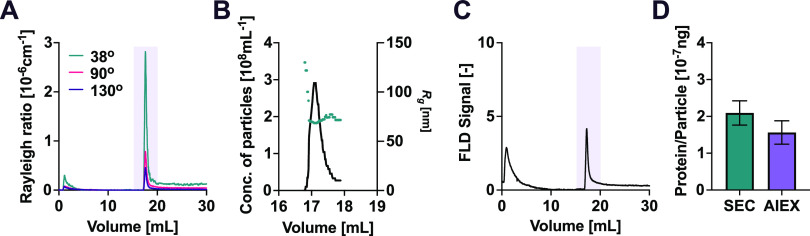
Using anion exchange chromatography (AIEX) to characterize the impurities in a SEC-purified EV sample. (A) Measured scattered light intensity (Rayleigh ratio) after AIEX at different elution volumes at selected detectors (38, 90, and 130° angles). (B) Measured EV radius of gyration (green dots) and concentrations (black line) at different elution volumes corresponding to the area highlighted in purple in panel (A). (C) Intrinsic fluorescence chromatograph after separation by AIEX. The area highlighted in purple corresponds to the elution of EVs. (D) Comparison of ng protein per particle after SEC and after additional purification by AIEX.

To illustrate an example of this approach, we applied anion exchange chromatography (AIEX) to further fractionate an EV sample that was previously purified by SEC. We analyzed the eluate simultaneously with MALS (Figure 7A,B) and FLD (Figure 7C). The chromatogram showed the presence of two peaks: one main peak eluting after 17 mL and corresponding to EVs^26^ with an average diameter of 151 nm (Figure 7B) and a second smaller peak eluting after 1 mL. This second peak contains proteins (Figure 7C) and exhibits weak scattering (Figure 7A), indicating that this fraction represents protein impurities that would co-elute with EVs when purified by SEC. As further confirmation, the protein-to-particle ratio of EVs decreased after ion exchange purification (Figure 7D).

In summary, the combination of liquid chromatography with MALS-FLD was able to characterize the number and size of particles, the total protein amount, and the presence of CD81+ particles from 20 μL of conditioned media, as well as characterize the purity of an EV sample by separating contaminants by using AIEX.

## Conclusions

We have described the use of liquid chromatography coupled to in-line multi-angle light scattering (MALS) and fluorescence detection (FLD) for the characterization of EVs. With this combination, many EV attributes required by the MISEV2018 guidelines have been characterized.

MALS detection provides the average radius of gyration and number of particles, with a limit of detection of at least 10^7^ particles. The sensitivity and precision of the analysis can be further improved in the future by a more accurate estimation of the effective refractive index of EV, which depends also on the components such as proteins and nucleic acids inside the lumen,^30−33^ and of the form factor.

Measurement of intrinsic protein fluorescence enables the label-free and non-destructive measurement of the total protein amount and, when combined with MALS, the protein-to-particle ratio.

Moreover, by labeling the EVs with suitable strategies, fluorescence detection can report on the presence of components such as EV-specific surface markers and lipid membranes, as well as confirm the presence of hollow morphology. This analysis can be expanded in the future with other fluorescent markers to characterize, for instance, labeled nucleotides or fluorescent cargos.

Finally, the combination of these in-line detectors with orthogonal chromatographic methods (based, for instance, on size and surface charge) enables the analysis of sample purity and of EVs directly from crude mixtures such as conditioned media.

The method does not perform single-particle analysis and cannot replace the battery of biophysical tools required for a comprehensive characterization of complex EV samples. However, this versatile approach offers a good compromise between the quantity of information and the amount of sample, time, and effort required by the analysis. In analogy with a microfluidic format previously developed in our group,^11^ the core of the approach is the multiparametric analysis of the EV sample on the same platform. Coupling the orthogonal detections with a fractionation method extends the analysis to more complex mixtures and allows to evaluate the EV sample purity.

We envision that this analytical approach can be useful for the optimization and quality control of EV manufacturing and engineering, e.g., upstream and downstream operation units and fluorescent cargo loading, as well as in the analysis of biological fluids, e.g., blood,^34,35^ due to the low sample consumption, compatibility with complex matrices, and high-throughput.

## Supporting Information Available

Derivation of eq 3 and comparison of average sizes from MALS, NTA, and DLS (DOCX)
