# Supplementary material for: Multiparametric Orthogonal Characterization of Extracellular Vesicles by Liquid Chromatography Combined with In-Line Light Scattering and Fluorescence Detection
Source: Anal Chem. 2023 Aug 9;95(33):12443–51. doi: 10.1021/acs.analchem.3c02108 (PMC10448444; doi:10.1021/acs.analchem.3c02108)
Supplement: Supplementary file 1 — ac3c02108_si_001.pdf [file ac3c02108_si_001.pdf]

Supporting Information

Multi-parametric orthogonal characterization of extracellular vesicles by liquid chromatography combined with in-line light scattering and fluorescence detection

Karl Normak[a], Marcell Papp[a], Michael Ullmann[a], Carolina Paganini[a], Mauro Manno[b], Antonella Bongiovanni[c], Paolo Bergese[d], Paolo Arosio[a]

[a] Department of Chemistry and Applied Biosciences, ETH Zurich, Zurich, Switzerland
[b] Institute of Biophysics, National Research Council of Italy, Via Ugo la Malfa 153, Palermo 90146, Italy
[c] Institute for Research and Biomedical Innovation (IRIB), National Research Council of Italy, Via Ugo La Malfa 153, Palermo, 90146, Italy
[d] Department of Molecular and Translational Medicine, University of Brescia, Brescia, 25123, Italy, Center for Colloid and Surface Science (CSGI), Florence, 50019, Italy

Abstract
In this supporting information we describe in more detail how to calculate the number of particles by light scattering. We also compare the average particle size measured by different techniques and show the agreement between MALS, NTA and DLS.

Table of contents

	Page
Determination of particle concentration from light scattering intensity	S-2
Figure S2 Comparison of average sizes measured by the MALS, NTA and DLS.	S-4

Determination of particle concentration from light scattering intensity

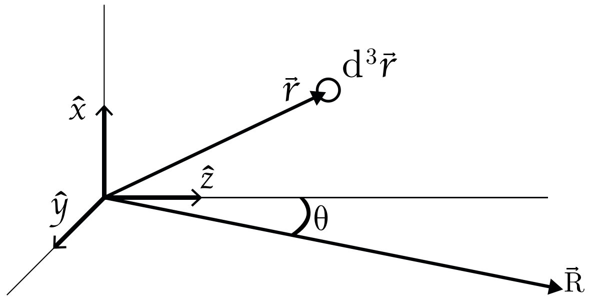

Figure S1 Scheme of the light scattering

We consider an incident electric field $\vec{E_i}$ with wavelength λ polarized along $\hat{x}$ and propagating along $\hat{z}$ in a medium with refractive index $n_0 = \sqrt{\epsilon_0}$ and $\vec{k_i} = 2\pi n_0 \lambda^{-1}$:

$$\vec{E_i}(\vec{r},t) = \hat{x}E_0 e^{-i\omega t + i\vec{k_i}\cdot\vec{r}}$$

The scattered electric field $\vec{E}_s$ at the point $\vec{R}$ (e.g. the detector) by a scattering volume V_s is:

$$\vec{E}_s(\vec{R},t) = \nabla \vee \nabla \vee \int_{V_s} \frac{1}{|\vec{R}-\vec{r}|} \overrightarrow{\overrightarrow{\delta_\epsilon}}(\vec{r},t') \cdot \vec{E}(\vec{r},t') d^3\vec{r}$$

Where $\overrightarrow{\overrightarrow{\delta_\epsilon}}(\vec{r},t')$ and $\vec{E}(\vec{r},t')$ are the local dielectric constant fluctuation tensor and the electric field at point $\vec{r}$ and at the retarded time $t' = t - \frac{n_0}{c}|\vec{R}-\vec{r}|$

Taking the following assumptions:

1) Elastic scattering - The frequency of the scattering and incident field are equal
2) Born approximation – the electric field in V_s is equal to the incident field: $\vec{E}(\vec{r},t') = \vec{E_i}(\vec{r},t')$
3) Fraunhofer approximation – the detector is far from the scattering medium and implies that $|\vec{R}-\vec{r}| \approx R - \vec{r}\cdot\vec{R}R^{-1}$ and, therefore:

$$\omega t' = \omega t - k|\vec{R} - \vec{r}| = \omega t - kR + \vec{r}\cdot\overrightarrow{k_s}$$

, where $k = \frac{2\pi n_0}{\lambda}$ and $\overrightarrow{k_s} = k\frac{\vec{R}}{R}$ is a vector pointing in the scattering direction. Defining the scattering vector as $\vec{q} = \overrightarrow{k_0} - \overrightarrow{k_s}$, we obtain:

$$\vec{E}_s(\vec{R},t) = \frac{E_0}{R} e^{-i\omega t + ikR} k_s \wedge k_s \wedge \int_{V_s} e^{i\vec{q}\cdot\vec{r}} [\overrightarrow{\overrightarrow{\delta_\epsilon}}(\vec{r},t') \cdot \hat{x}] d^3\vec{r}$$

Taking the further assumptions:

1) A symmetric and isotropic dielectric constant: $\overrightarrow{\overrightarrow{\delta_\epsilon}}(\vec{r},t') = \delta_\epsilon(\vec{r},t)\vec{\vec{I}}$
2) The detector lies in the *yz* plane for simplicity

then, the scattered field is polarized along $\hat{x}$ and depends on the angle θ between the incident light and the scattered light. Therefore, the scattering vector $q = |\vec{q}| = 2k\sin\left(\frac{\theta}{2}\right)$.
By introducing the spatial Fourier transform of the dielectric constant fluctuation

$$\delta_\epsilon(\vec{q},t) = \int_{V_s} e^{i\vec{q}\cdot\vec{r}}\, \delta_\epsilon(\vec{r},t') d^3\vec{r}$$

the electric field scattered can be expressed in terms of the scattering vector:

$$E_s(q,t) = \frac{E_0 k^2}{R}\, e^{-i\omega t + ikR} \delta_\epsilon(\vec{q},t)$$

The measurable normalized quantity of light scattering intensity is the Rayleigh ratio:

$$R(q) = \frac{|E_s(q)|^2}{E_0^2}\frac{R^2}{V_s} = k^4 V_s^{-1}|\delta_\epsilon(\vec{q})|^2$$

For a homogeneous particle of volume V_p, refractive index n_p, and dielectric constant fluctuation $\delta_{\epsilon_p} = \frac{n_p^2 - n_0^2}{4\pi n_0^2}$

$$\delta_\epsilon(\vec{q}) = \delta_{\epsilon_p}\int_{V_s} e^{i\vec{q}\cdot\vec{r}} = \delta_{\epsilon_p} V_p f_p(q) = \frac{n_p^2 - n_0^2}{4\pi n_0^2} V_p f_p(q)$$

where $f_p(q)$ is the form amplitude

Since the Born approximation taken before is reasonable for $n_p - n_0 \ll n_0$ we can further approximate:

$$\delta_{\epsilon_p} = \frac{n_p^2 - n_0^2}{4\pi n_0^2} \approx \frac{2n_0(n_p - n_0)}{4\pi n_0^2} = \frac{n_p - n_0}{2\pi n_0}$$

If in the scattering volume V_s there are c number concentration of homogeneous, non-interacting particles, the scattering intensity is:

$$R(q) = k^4 c\left[\frac{n_p - n_0}{2\pi n_0}\right]^2 V_p^2 P(q)$$

where $P(q) = \left|f_p(q)\right|^2$ is the form factor.

References:

1. Berne, Bruce J., and Robert Pecora. Dynamic Light Scattering: with Applications to Chemistry, Biology, and Physics. Wiley, 1976.
2. Purcell, Edward M. Electricity and Magnetism. McGraw-Hill, 1965.
3. Kerker, Milton. The Scattering of Light and Other Electromagnetic Radiation. Academic Press, 1969.

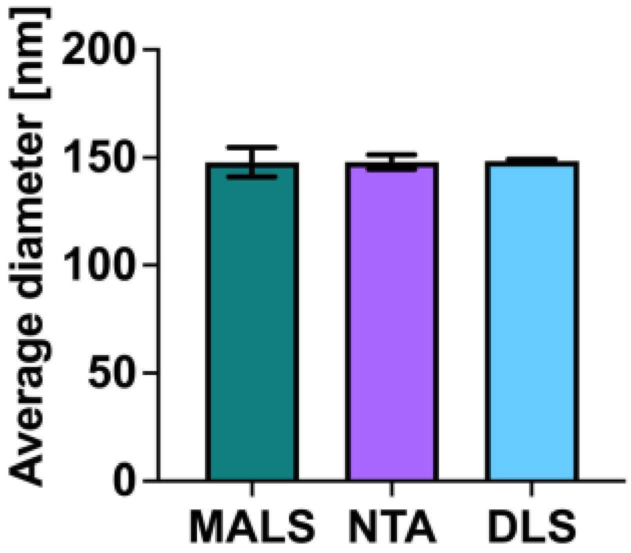

Figure S2. Comparison of average sizes measured by the MALS, NTA and DLS.
